# Moving from nature to nurture: a systematic review and meta-analysis of environmental factors associated with juvenile idiopathic arthritis

**DOI:** 10.1093/rheumatology/keab627

**Published:** 2021-08-11

**Authors:** Sarah L N Clarke, Katie S Mageean, Ilaria Maccora, Sean Harrison, Gabriele Simonini, Gemma C Sharp, Caroline L Relton, Athimalaipet V Ramanan

**Affiliations:** MRC Integrative Epidemiology Unit; Population Health Sciences, Bristol Medical School, University of Bristol; Department of Paediatric Rheumatology, University Hospitals Bristol NHS Foundation Trust, Bristol, UK; Department of Paediatric Rheumatology, University Hospitals Bristol NHS Foundation Trust, Bristol, UK; Rheumatology Unit, A Meyer Children Hospital, NEUROFARBA Department, University of Florence, Florence, Italy; MRC Integrative Epidemiology Unit; Population Health Sciences, Bristol Medical School, University of Bristol; Rheumatology Unit, A Meyer Children Hospital, NEUROFARBA Department, University of Florence, Florence, Italy; MRC Integrative Epidemiology Unit; Population Health Sciences, Bristol Medical School, University of Bristol; MRC Integrative Epidemiology Unit; Population Health Sciences, Bristol Medical School, University of Bristol; Department of Paediatric Rheumatology, University Hospitals Bristol NHS Foundation Trust, Bristol, UK; Translational Health Sciences, Bristol Medical School, University of Bristol, Bristol, UK

**Keywords:** juvenile idiopathic arthritis, risk factor, environmental

## Abstract

**Objectives:**

JIA is the most common paediatric rheumatic disease, thought to be influenced by both genetics and the environment. Identifying environmental factors associated with disease risk will improve knowledge of disease mechanism and ultimately benefit patients. This review aimed to collate and synthesize the current evidence of environmental factors associated with JIA.

**Methods:**

Four databases (MEDLINE, Embase, Web of Science and Cumulative Index to Nursing and Allied Health Literature) were searched from inception to January 2020. Study quality was rated using the Newcastle-Ottawa Scale. Pooled estimates for each environmental factor were generated using a random-effects, inverse-variance method, where possible. The remaining environmental factors were synthesized in narrative form.

**Results:**

This review includes 66 environmental factors from 39 studies (11 cohort and 28 case-control studies) over 45 years. Study sample sizes ranged from 41 to 1.9 million participants. Eight environmental factors from ten studies were meta-analysed. Caesarean section delivery was associated with increased JIA risk [pooled odds ratio (OR) 1.11, 95% CI: 1.01, 1.22]. Conversely, presence (*vs* absence) of siblings (pooled OR 0.60, 95% CI: 0.44, 0.81) and maternal prenatal smoking (pooled OR 0.70, 95% CI: 0.58, 0.84) were associated with decreased JIA risk.

**Conclusion:**

This review identifies several environmental factors associated with JIA and demonstrates the huge breadth of environmental research undertaken over five decades. We also highlight the challenges of combining data collected over this period due to limited between study comparability, evolution in healthcare and social practices, and changing environment, which warrant consideration when planning future studies.

**Rheumatology key messages**

- Despite being a complex disease, robust evidence of environmental associations of JIA incidence is limited.
- We identified strong evidence of environmental factors associated with both increased and decreased JIA risk.
- The findings of this study will inform (causal) research and contribute to understanding JIA aetiopathogenesis.

## Introduction

JIA is the most common rheumatic disease of childhood with a pooled prevalence of 32.6/100 000 in Caucasians [1]. International consensus criteria currently define seven subtypes of JIA based on their clinical features [2]; however, diagnostic criteria have evolved over the years [3]. JIA is considered a complex disease influenced by both non-Mendelian genetics and the environment. The genetic contribution to JIA, examined both in familial studies and in genome-wide association studies, has been estimated to be between 13% and 32% [4–7]. A substantial contribution to disease risk is likely attributed to gene-environment interactions and environmental factors.

Studies of other autoimmune diseases demonstrate the potential for environmental exposures to induce epigenetic changes [8], modulate the immune system [9] or alter the microbiome [10]. While the role of environmental factors in JIA is less clear, it is likely that similar mechanisms influence JIA risk. Two narrative reviews of environmental factors associated with JIA have been published in recent years [11, 12] that highlight several key limitations of research to date. Firstly, the availability of high-quality data in JIA is limited, often of modest sample size and lags behind that of other autoimmune conditions. Secondly, many environmental factors studied so far, such as breastfeeding, infection and smoking, require further scrutiny. Finally, several environmental factors, such as diet, remain relatively unexplored.

Identifying environmental factors associated with JIA, and quantifying their effects, has the potential to benefit patients by guiding research priorities and informing future studies probing causality and disease mechanism. Such developments could inform new therapeutic modalities and assist in patient counselling and risk stratification. Finally, environmental associations may be readily and/or feasibly modifiable, which could translate into lower disease burden within populations.

The aim of this systematic review and meta-analysis was to collate, quantify and evaluate the current evidence for environmental factors that influence JIA risk and highlight areas of unmet research need.

## Methods

This review follows guidance from the Preferred Reporting Items for Systematic Reviews and Meta-analyses (PRISMA) statement [13] and the protocol was pre-registered on PROSPERO (ID: CRD42017078306) [14].

### Literature searching

Expert advice from a literature searching specialist was sought prior to designing the search strategy. A systematic search of MEDLINE (Ovid), Embase (Ovid), Cumulative Index to Nursing and Allied Health Literature (CINAHL, EBSCOhost) and Web of Science (WOS, Clarivate Analytics) was performed, with English language restriction, from database inception to 18 January 2020. The MEDLINE search strategy (Table 1) was amended for use in other databases (Supplementary Table S1, available at *Rheumatology* online). Bibliographies of excluded review articles were hand-searched to identify further relevant studies.

**Table 1:** Search syntax used for MEDLINE, amended for use in other bibliographic databases

Line	Syntax
1	(juvenile adj2 arthritis).tw
2	Arthritis, Juvenile/
3	Risk Factors/
4	Environment/
5	Seasons/
6	Postpartum Period/
7	Pregnancy/
8	Birth order/
9	age factors/or maternal age/
10	paternal age/
11	Socioeconomic Factors/
12	Demography/
13	Infection/
14	Communicable Diseases/
15	Bacterial Infections/
16	risk.tw
17	environmen*.tw
18	perinatal.tw
19	(season* adj3 birth).tw
20	smok*.tw
21	1 or 2
22	3 or 4 or 5 or 6 or 7 or 8 or 9 or 10 or 11 or 12 or 13 or 14 or 15 or 16 or 17 or 18 or 19 or 20
23	21 and 22

### Study selection

All aspects of study selection and risk of bias assessment were undertaken independently by two reviewers (S.L.N.C. and either K.S.M. or I.M.). Any discrepancies were resolved by discussion then involvement of an additional reviewer (A.V.R.). References were downloaded into Endnote (version X9.3.2, Clarivate Analytics) and de-duplicated as recommended [15]. Blind title and abstract screening was performed using Rayyan (QCRI) [16] against the inclusion/exclusion criteria (Table 2). The full texts of potentially relevant studies were retrieved and further reviewed against the inclusion/exclusion criteria.

**Table 2:** Inclusion and exclusion criteria for studies

Domain	Inclusion criteria	Exclusion criteria
Study language	English	Not English
Study type	Systematic reviews	Review articles (non-systematic)
	Observation studies (cohort, case-control, cross-sectional)	Clinical trials
		Animal studies
		*In vitro* studies
		*Ex vivo* studies
		Case reports
Study population	Patient with JIA [as diagnosed using any recognized criteria, e.g. ACR, EULAR, International League Against Rheumatism (ILAR)]	Adults (defined as age >16yrs)
Study comparator	General population without JIA or JIA-U	Other rheumatic, autoimmune or inflammatory disease
Study risk factor	Environmental risk factors (including patient, familial and perinatal)	Non-environmental risk factors (e.g. genetic, ethnic/racial, familial aggregation)

### Data extraction

Data items (Supplementary Table S2, available at *Rheumatology* online) were extracted from each included study by S.L.N.C. into a predesigned template. This was independently checked by either K.S.M. or I.M., with involvement of an additional reviewer (A.V.R.) where necessary. Where a study included multiple case or control groups, data was extracted from (i) the most matched case and control groups and (ii) community control groups in preference to hospital control groups. Corresponding authors were contacted to obtain missing data or clarify data ambiguities.

### Risk of bias (quality) assessment

We used the Newcastle-Ottawa Scale (NOS) for case-control and cohort studies to assess the risk of bias and methodological quality of included studies [17]. The scale scores three study domains (selection criteria, comparability and outcome) with a maximum score of nine indicating the highest quality. A risk of biases table was created, summarizing study quality.

### Data synthesis and statistical analysis

Data was analysed using the statistical software R (version 3.6.1) in RStudio. The data was synthesized into ten environmental factor ‘domains’ (maternal, paternal, perinatal, early life, smoking, environmental, socioeconomic, living environment, dietary and infections). For consistency, unadjusted odds ratios (OR) and 95% CI were derived for all variables where sufficient raw data was available. To aid synthesis, the data was condensed in two ways. Firstly, although all relevant data reported by a study was extracted, only the most inclusive evidence for each environmental factor from each study was included in our synthesis, as jointly determined by S.L.N.C. and A.V.R. For example, sex-stratified or JIA subtype specific analyses were not included in the narrative synthesis where a mixed-sex or pan-JIA subtype analysis, respectively, was reported. Secondly, a single point estimate with 95% CIs was reported for each variable from each study. This consisted of, in order of priority and subject to data availability, the most adjusted point estimate, the derived unadjusted estimate or the study reported unadjusted estimate. Our complete dataset is included in Supplementary Table S3, available at *Rheumatology* online, illustrating the data inclusion decisions. Forest plots were constructed summarizing a single point estimate with 95% CI for each environmental factor. Pooled ORs were generated for environmental factors examined in multiple studies in a comparable manner (clinically and statistically). Where possible, ordinal categorical data was converted to a single estimate using generalized least squares of trend [18] implemented in the *glst* module [19] in Stata (version 14.2) prior to meta-analysis (Supplementary Data S1, available at *Rheumatology* online). Only studies reporting adjusted estimates were included in meta-analysis. All meta-analyses used the inverse weighted variance method in the *meta* R package using a random effects model. Statistical heterogeneity was assessed using Cochran’s Q test (χ^2^ test) and Higgin’s I^2^ value [20].

## Results

### Study selection

**Fig. 1:**
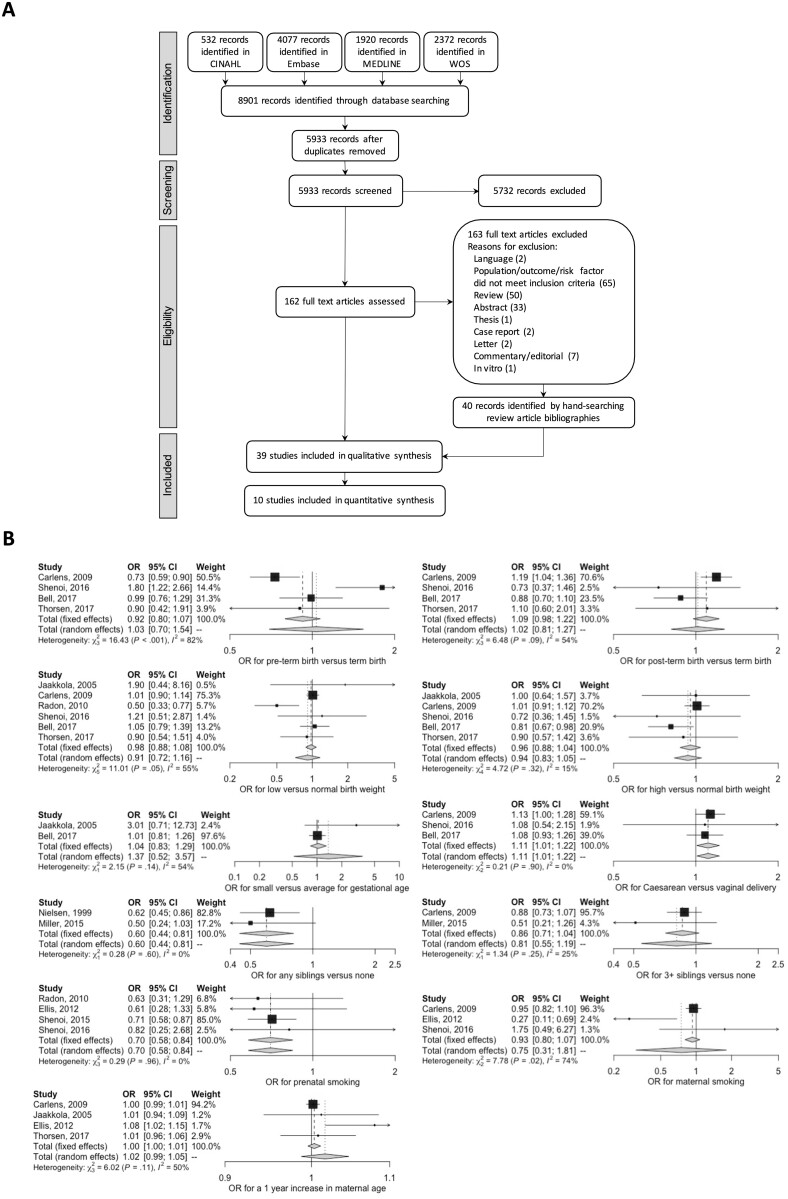
PRISMA flow diagram for study selection (**A**) and results of meta-analysis (**B**) (**A**) PRISMA flow diagram showing the selection of studies included in this review. CINAHL: Cumulative Index to Nursing and Allied Health Literature; PRISMA: Preferred Reporting Items for Systematic reviews and Meta-Analyses; WOS, Web of Science. (**B**) Meta-analysis results of studies examining associations between JIA and perinatal, early life, maternal and smoking related factors. OR: odds ratio.

Literature searches identified 5933 unique database records (Fig. 1A). The majority (5731/5933) were excluded during title and abstract screening. Of the 202 full-text records examined, 39 studies met the pre-specified inclusion criteria.

### Study characteristics

Table 3 details the 11 cohort and 28 case-control studies included in this review. Studies originate from 13 countries and were published between 1974 and 2019. Sample sizes ranged from 41 to 1 900 000 participants. JIA cases from mixed-sex studies were predominantly female (range 52.6–81.5% female); four studies were restricted to single-sex participants [21–24]. Studies differed considerably in overall design, encompassing nine different versions of JIA diagnostic criteria and variable inclusivity of JIA subtypes. Most studies (23/39) undertook covariate adjustment and 20/28 case-control studies matched cases and controls.

**Table 3:** Characteristics and design of included studies

Study	Study type	Country	Sample size	Age range (years)	Sex (female, %)	JIA criteria used ^c^	JIA subtypes included^c^	Risk factors examined	Matching of controls
Cassidy, 1974 [54]	Case-control	USA	131 cases/80 controls	2–18	66.4	ACR 1973	No exclusions reported	Rubella, rubeola	No
Chantler, 1985 [55]	Case-control	Canada	19 cases/8 controls	8–20	52.6	ACR 1977	No exclusions reported	Rubella	No
Kunnamo, 1987 [46]	Case-control	Finland	26 cases/26 controls	<16 years		ACR 1977	No exclusions reported	Upper respiratory tract infection, daycare	Date of birth, sex, geography
Mason, 1995 [43]	Case-control	USA	54 cases/79 controls	5.4 (±3.8)^a^	81.5	ACR 1977	Excluded spondyloarthropathies	Breastfeeding	Age, race
Tsai, 1995 [53]	Case-control	China	21 cases/20 controls	13^a^		ACR 1977	No exclusions reported	CMV, EBV	Age
Rosenberg, 1996 [44]	Case-control	Canada	137 cases/331 controls			ACR 1977	No exclusions reported (no systemics included)	Breastfeeding, cow's milk	No (parent selected)
Nielsen, 1999 [37]	Case-control	Denmark	220 cases/880 controls	6^b^		EULAR 1978	Excluded spondyloarthropathies	Siblings, income, residential factors, parental employment/occupation, season of birth	Age, sex, geography
Angelini, 2003 [50]	Case-control	Italy	35 cases/93 controls	2.0–14	75.6	ACR 1987	All	Parvovirus B19	Sex
Lehmann, 2003 [51]	Case-control	Germany	68 cases/124 controls			ILAR 1997	No exclusions reported	Parvovirus B19	Age
Prahalad, 2003 [40]	Case-control	USA	333 cases/3295 controls	61.86	61.9	ILAR 1997	No exclusions reported	Siblings, birth order	Sex, birth year
Altun, 2004 [48]	Case-control	Turkey	60 cases/35 controls	1.16–15.6	68.3	ILAR 1997	Excluded ERA	Chlamydophila pneumoniae	Age
Jaakkola, 2005 [25]	Retrospective cohort	Finland	58 841		74.2	ICD-9	Code 714.3	Birth weight, size for gestational age, maternal age, parity, maternal civil status, parental employment/occupation, maternal prenatal smoking	n/a
Gonzalez, 2007 [52]	Case-control	Chile	50 cases/39 controls	0.5–12	60.0	ILAR 1997, EULAR 1978, ACR 1977	No exclusions reported	Parvovirus B19	No
Carlens, 2009 [26]	Case-control	Sweden	3334 cases/13 336 controls	3^b^	54.0	ICD8-10	ICD codes not listed	Maternal age, maternal civil status, siblings, season of birth, mode of delivery, type of birth, birth weight, size for gestational age, gestational age, congenital malformation, maternal smoking, apgar score at 5 min, infections in first year of life	Age, sex, hospital of birth
Radon, 2010 [34]	Case-control	Germany	238 cases/832 controls	10.9 (±3.1)^a^	71.4	ILAR 2001	Oligo only	Parental education, breastfeeding, birth weight, maternal prenatal smoking, residential factors, daycare, allergy, pets/animals	No
Aslan, 2011 [49]	Case-control	Turkey	26 cases/24 controls	1.58–4.58	69.2	ILAR 1997	Excluded ERA and HLA-B27+ patients	*Mycoplasma pneumoniae*, *Borrelia burgdorferi*, *Chlamydophila pneumoniae*, *Chlamydia trachomatis*, *Campylobacter jejuni*, EBV, Parvovirus B19, culture positive	No
Bager, 2012 [56]	Retrospective cohort	Denmark	924 749			ICD-10	Codes M08.0, M08.2, M08.3, M08.4, M08.8, M08.9	Mebendazole	n/a
Ellis, 2012 [27]	Case-control	Australia	229 cases/458 controls	9.4 (±4.6)^a^	68.1	ILAR 2001	No exclusions reported	Socioeconomic status, parental education, maternal civil status, parental employment/occupation, maternal smoking, maternal age, maternal prenatal smoking, diet/supplement use during pregnancy, paternal civil status, paternal smoking, paternal age, paternal prenatal smoking, breastfeeding, cow's milk, mode of delivery	Cases matched to geographical controls
Neufeld, 2013 [41]	Case-control	Canada	373 cases/987 controls	8.1 (male)^a^, 10.7 (female)^a^	66.2	ILAR 2001, ACR 1977	Excluded psoriatic and IBD related	Stressful life events, residential factors	Age, sex, geography
Arnheim-Dahlstrom, 2013 [22]	Retrospective cohort	Denmark, Sweden		10–17	100.0	ICD-10	ICD codes M08.0, M08.2-9	HPV vaccination	n/a
Arvonen, 2015 [58]	Case-control	Finland	1298 cases/5179 controls	0.8–12.9	63.3	ICD-10	ICD code M08	Antibiotic use	Age, sex, geography
Berkun, 2015 [38]	Case-control	Israel	558 cases/1 040 558 controls		67.6	ILAR 2001	All	Season of birth	No
Sevelsted, 2015 [35]	Retrospective cohort	Denmark	1 900 000			ICD-8 and ICD-10	Codes 712.00 (ICD-8) and M05.x, M08x, M09x, M13.x (ICD-10)	Mode of delivery	n/a
Miller, 2015 [39]	Case-control	Australia	302 cases/341 controls	8.5 (±4.7)^a^	66.9	ILAR 2001	Excluded systemics	Birth order, siblings	Geography
Shenoi, 2015 [47]	Case-control	USA	1196 cases/5618 controls	<16 years	67.7	ILAR 2001	No exclusions reported	Maternal prenatal smoking	Birth year, geography
Haerskjold, 2016 [57]	Retrospective cohort	Denmark, Sweden	1 351 265			ICD-10	Codes M08, M09	Palivizumab	n/a
Kristensen, 2016 [36]	Retrospective cohort	Denmark	790 569	0–14		ICD-10	Codes M08.0-M08.9	Mode of delivery	n/a
Shenoi, 2016 [28]	Case-control	USA	225 cases/138 controls	<16 years	70.7	ILAR 2001	No exclusions reported	Income, birth weight, mode of delivery, gestational age, BMI at time of survey, household smoking, maternal prenatal smoking, duration prenatal smoking, months, maternal smoking, paternal smoking, relative smoking, breastfeeding, cow's milk, infections in first year of life, daycare, birth order, stressful life events, maternal age, paternal age, maternal civil status, residential factors, pets/animals	Age, sex
Arvonen, 2017 [42]	Case-control	Finland	1298 cases/5179 controls	0.7–13	63.3	ICD-10, ILAR 2001	ICD10 codes M08	Cow's milk, antibiotic use, combined effect of cow's milk allergy and antibiotics	4:1 based on age, sex, municipality of residence
Bell, 2017 [30]	Case-control	USA	1252 cases/6072 controls	<16 years	67.3	ILAR 2001	No exclusions reported	Type of birth, gestational age, size for gestational age, birth weight, mode of delivery, no of prior births, foetal loss	Birth year, geography
Kindgren, 2017 [45]	Case-control	Sweden	32 cases/10 883 controls			ICD8-10	Codes M08-09	Breastfeeding, cow's milk, introduction of gluten	No
Thorsen, 2017 [29]	Case-control	Denmark	300 cases/300 controls	3–9	69.7	ILAR 1997, ILAR 2001, EULAR 1978	Included oligo and poly only (RF+/−)	Vitamin D, gestational age, birth weight, maternal age	Date of birth
Franca, 2018 [32]	Case-control	Brazil	66 cases/124 controls	10.8 (±3.9)^a^	59.1	ILAR 2001	No exclusions reported	Socioeconomic status, prenatal pollution exposure, maternal prenatal smoking, maternal weight gain in pregnancy, diet/supplement use during pregnancy, gestational age, birth weight, season of pregnancy, smoke exposure, daycare, pollution exposure	Age, sex
Skufca, 2018 [21]	Retrospective cohort	Finland	240 605		100.0	ICD-10	ICD code M080, M082, M083, M084, M089	HPV vaccination	n/a
Frisch, 2018 [24]	Retrospective cohort	Denmark	568 410		0.0	ICD-10	ICD codes M08.0, M08.2-9	HPV vaccination	n/a
Liu, 2018 [23]	Retrospective cohort	Canada	290 939		100.0	ICD-9, ICD-10	ICD-9 codes 711, 714, 715 and ICD-10 codes M05, M06, M08	HPV vaccination	n/a
Chiaroni-Clarke, 2019 [59]	Case-control	Australia	202 cases/202 controls	7.38 (±4.29)^a^	72.0	ILAR 2001	No exclusions reported	Lifetime sun exposure, prenatal sun exposure	Geography, birth year, time of recruitment
Kindgren, 2019 [33]	Prospective cohort	Sweden	15 771			ICD-9, ICD-10	ICD codes M08-09	Diet/supplement use during pregnancy, fish, heavy metals	n/a
Sperling, 2019 [31]	Retrospective cohort	Denmark	1 084 184	6.3 (3.1–10.6)^b^		ICD-10	ICD codes M08	Fertility problems	n/a

a mean (s.d.). ^b^median (interquartile range). ^c^details of the JIA criteria and ICD codes listed can be found in Supplementary Table S4, available at *Rheumatology* online. ERA: enthesitis-related arthritis; HPV: human papilloma virus; ICD: International Classification of Diseases; ILAR: International League of Associations for Rheumatology.

### Risk of bias

Included studies were of variable quality; NOS scores ranged from two to nine. Cohort studies were of higher quality than case-control studies overall (mean NOS score 6.6 *vs* 4.4, Tables 4 and 5). Scores were commonly downgraded for lack of independent validation of case status (case-control studies), inadequacy of follow up (cohort studies) and limited confounder adjustment.

**Table 4:** Newcastle-Ottawa Scale risk of bias assessment scores for case-control studies

Study	Selection				Comparability		Exposure				Total
	Adequate case definition with independent validation	Cases obviously representative	Selection of community control participants	Controls have no history of disease outcome	Study controls for socio economic status	Study controls for maternal age	Source of ascertainment		Same method of ascertain for cases and controls	Non response rate same for both groups	
							Secure record	Blinded interview			
Cassidy, 1974 [54]	0	0	1	1	0	0	1	0	1	1	5
Chantler, 1985 [55]	0	0	0	0	0	0	1	0	1	1	3
Kunnamo, 1987 [46]	1	1	1	0	0	0	0	0	1	0	4
Tsai, 1995 [53]	0	0	0	1	0	0	1	0	0	0	2
Mason, 1995 [43]	0	0	1	0	0	0	0	0	1	0	2
Rosenberg, 1996 [44]	0	1	1	1	0	0	0	0	1	0	4
Nielsen, 1999 [37]	1	1	1	0	1	0	1	0	1	0	6
Angelini, 2003 [50]	0	0	0	1	0	0	1	0	1	1	4
Lehmann, 2003 [51]	0	1	0	0	0	0	1	0	1	1	4
Prahalad, 2003 [40]	1	0	1	0	0	0	0	0	1	1	4
Altun, 2004 [48]	0	0	0	1	0	0	1	0	1	1	4
Gonzalez, 2007 [52]	1	1	0	1	0	0	1	0	1	1	6
Carlens, 2009 [26]	0	1	1	0	0	1	1	0	1	1	6
Radon, 2010 [34]	0	1	0	0	1	0	0	0	1	1	4
Aslan, 2011 [49]	0	0	0	1	0	0	1	0	1	1	4
Ellis, 2012 [27]	0	0	0	0	1	1	0	0	1	0	3
Neufeld, 2013 [41]	0	1	1	0	0	0	0	0	1	0	3
Arvonen, 2015 [58]	0	1	1	1	0	0	1	0	1	1	6
Berkun, 2015 [38]	0	0	1	0	0	0	0	0	0	0	1
Miller, 2015 [39]	0	0	1	1	0	1	0	0	1	0	4
Shenoi, 2015 [47]	1	0	1	0	0	1	0	0	1	1	5
Shenoi, 2016 [28]	0	0	1	1	1	0	0	0	1	0	4
Arvonen, 2017 [42]	0	1	1	1	0	0	1	0	1	1	6
Bell, 2017 [30]	1	0	1	0	0	1	1	0	1	1	6
Kindgren, 2017 [45]	1	1	1	0	1	1	0	0	1	0	6
Thorsen, 2017 [29]	0	0	1	0	0	1	1	0	1	0	4
Franca, 2018 [32]	0	1	1	1	1	1	0	0	1	0	6
Chiaroni-Clarke, 2019 [59]	1	1	0	1	1	1	0	0	1	0	6

**Table 5:** Newcastle-Ottawa Scale risk of bias assessment scores for cohort studies

Study ID	Selection						Comparability		Outcome					Total
	Represen tativeness of exposed cohort		Non-exposed cohort from same community as exposed cohort	Source of ascertainment		Outcome of interest not present at start of study	Study controls for socio economic status	Study controls for maternal age	Assessment of outcome		Follow up long enough for outcome to occur	Adequacy of follow up		
	Truly	Somewhat		Secure recorded	Blinded interview				Independent blind assessment	Record linkage		Complete follow up	>95% follow up or description provided of loss to follow-up	
Jaakkola, 2005 [25]	1	0	1	1	0	1	1	1	0	1	0	0	1	8
Bager, 2012 [56]	1	0	1	1	0	1	1	1	0	1	0	0	0	7
Arnheim- Dahlstrom, 2013 [22]	1	0	1	1	0	1	1	1	0	1	0	0	0	7
Sevelsted, 2015 [35]	0	1	1	1	0	1	0	1	0	1	0	0	0	6
Haerskjold, 2016 [57]	1	0	1	1	0	1	0	0	0	1	0	0	0	5
Kristensen, 2016 [36]	0	1	1	1	0	1	0	1	0	1	0	0	0	6
Frisch, 2018 [24]	1	0	1	1	0	1	0	1	0	1	0	0	0	6
Liu, 2018 [23]	1	0	1	1	0	1	0	0	0	1	0	0	0	5
Skufca, 2018 [21]	1	0	1	1	0	1	0	0	0	1	0	0	0	5
Kindgren, 2019 [33]	1	0	1	1	0	1	1	1	0	1	0	0	1	8
Sperling, 2019 [31]	1	0	1	1	0	1	1	1	0	1	1	0	1	9

### Evidence synthesis

Sixty-six environmental factors were examined within this review of which half were only examined in a single study. Forty-five environmental factors had both covariate unadjusted and adjusted analyses; covariate unadjusted and adjusted data was available for 63 and 48 environmental factors, respectively. Eight environmental factors from ten studies were meta-analysed (Fig. 1B) with the remainder summarized in narrative form (Supplementary Fig. S1A–1J, available at *Rheumatology* online). Supplementary Table S3, also available at *Rheumatology* online, details all estimates extracted/derived for each study.

### Maternal factors

Maternal factors were examined in nine studies [25–33] (Supplementary Fig. S1A, available at *Rheumatology* online). Meta-analysis of four studies [25–27, 29] found no evidence of an association between JIA and maternal age (pooled OR 1.02, 95% CI: 0.99, 1.05, Fig. 1B). There was some evidence of a protective association between maternal multiparity and JIA [25, 30], albeit with imprecise estimates. One study [27] identified maternal civil status (married) as associated with increased JIA risk (OR 2.62, 95% CI: 1.16, 5.92), however this was unsupported by other studies [25, 26, 28]. Of the maternal dietary factors examined [27, 32, 33], only the consumption of fish more than once a week during pregnancy showed strong evidence of association with JIA (OR 4.50, 95% CI: 1.90, 10.40) in a single study [33]. Maternal fertility problems [31], but not prior foetal loss [30] or specific fertility treatment [31], was associated with increased JIA risk (hazard ratio (HR) 1.18, 95% CI: 1.05, 1.32).

### Paternal factors

Compared with maternal factors, there was little data regarding paternal factors associated with offspring JIA (Supplementary Fig. S1B, available at *Rheumatology* online). Two studies [27, 28] examining paternal age found no association with JIA. One study [27] found increased JIA risk associated with paternal civil status (OR 2.48, 95% CI: 1.06, 5.8), consistent with the corresponding maternal estimate.

### Perinatal factors

Perinatal factors were examined in 11 studies [25–30, 32, 34–37] (Supplementary Fig. S1C, available at *Rheumatology* online). Six perinatal factors were included in the meta-analysis: pre and post-term delivery, small size for gestational age (SGA), low and high birth weight (LBW and HBW) and Caesarean section delivery (CSD). The pooled estimate from three studies [26, 28, 30] identified strong evidence that CSD is a risk factor for JIA (OR 1.11, 95% CI: 1.01, 1.22). Sevelsted *et al.* [35] also reported increased JIA risk following CSD (adjusted incidence rate ratio (IRR) 1.10, 95% CI: 1.02, 1.18) as did Kristensen and Henriksen [36] for elective (adjusted HR 1.25, 95% CI: 1.04, 1.51) but not acute CSD (adjusted HR 0.99, 95% CI: 0.82, 1.20). Evidence of association between instrumental delivery and JIA was inconsistent [27, 28]. The pooled estimates from four studies [26, 28–30] found little evidence of an association between JIA and pre- or post-term delivery (OR 1.03, 95% CI: 0.70, 1.54 and OR 1.02, 95% CI: 0.81, 1.27 respectively). Little evidence of association was found between JIA and birth weight [25, 26, 28–30, 34] (pooled OR 0.91 95% CI: 0.71, 1.16 and OR 0.94 95% CI: 0.83, 1.05 for LBW and HBW, respectively). Similarly, there was little evidence of association between JIA and SGA [25, 30] (pooled OR 1.37, 95% CI: 0.52, 3.57). Evidence from single studies suggests that ideal or less than ideal maternal weight gain during pregnancy [32] and Apgar score <6 [26] at delivery are negatively associated with JIA. One study [38] reported a distinct pattern in month of birth of JIA patients (with peak in November to March) *vs* the general population but this was unsupported by other studies examining season of birth or pregnancy [26, 32, 37]. There was little evidence of association between JIA and multiple birth [26, 30], neonatal vitamin D levels [29] and congenital malformation [26].

### Early life factors

Five studies [26, 28, 37, 39, 40] examined the association between JIA and sibling-related factors (Supplementary Fig. S1D, available at *Rheumatology* online). Meta-analysis of two studies [37, 39] found strong evidence that having siblings was associated with decreased JIA risk (pooled OR 0.60, 95% CI: 0.44, 0.81). Though the association between JIA and ≥3 siblings was attenuated (pooled OR 0.81, 95% CI: 0.55, 1.19) [26, 39] and there was limited evidence of a dose-dependent association between JIA and sibling number [26, 39, 40]. Evidence of an association between JIA and birth order was inconsistent [28, 39, 40]. One study associated child obesity (BMI >30) with increased risk JIA (adjusted OR 3.98, 95% CI: 1.82, 8.34) but evidence was limited for other BMI categories [28]. Two studies examined the number and type of stressful life events [28, 41] with largely inconsistent estimates; for example, household unemployment [28] and maternal employment outside of the home [41] showed opposing effects (adjusted OR 0.23, 95% CI: 0.09, 0.59 and adjusted OR 0.65, 95% CI: 0.47, 0.90, respectively). The association between allergy and JIA was also conflicting; unadjusted OR 1.59, 95% CI: 1.19, 2.13 for cow’s milk allergy [42] and unadjusted OR 0.57, 95% CI: 0.34, 0.95 for allergic rhinitis [34].

### Dietary factors

Seven studies [27, 28, 33, 34, 43–45] examined dietary factors (Supplementary Fig. S1E, available at *Rheumatology* online). Breastfeeding was the most frequently investigated dietary factor but there is little evidence of a consistent association between breastfeeding status [27, 28, 43, 44] or duration [27, 28, 34, 43, 45] and JIA. Similarly, there is little evidence of association between JIA and age of introduction of cow’s milk [27, 28, 44, 45] or gluten [45]. One study associated the consumption of fish more than once a week during the first year of life with increased JIA risk (adjusted OR 5.10, 95% CI: 2.10, 12.40), in keeping with their maternal data [33].

### Living environment

Six studies [28, 32, 34, 37, 41, 46] examined factors associated with residential and family environment (Supplementary Fig. S1F, available at *Rheumatology* online). One study found day-care attendance was strongly associated with decreased JIA risk (adjusted OR 0.12, 95% CI: 0.04, 0.44) [32]; however, this was unsupported by others [28, 34, 46]. There was little consistent evidence of association between residential area and JIA; the strongly positive association between living in a flat (*vs* a farm) and JIA (adjusted OR 2.69, 95% CI: 1.19) [37] was unsupported by residential area data from other studies [28, 34, 41] or data regarding early life contact with animals/pets [28, 34].

### Socioeconomic factors

Socioeconomic factors were examined in six studies [25, 27, 28, 32, 34, 37] (Supplementary Fig. S1G, available at *Rheumatology* online). In single studies, JIA was associated with higher maternal education (adjusted OR 2.72, 95% CI: 1.19, 6.20) [27] and higher household income (adjusted OR 1.94, 95% CI: 1.23, 3.07) [37]; however, little evidence of association was seen between JIA and other socioeconomic factors.

### Smoking-related factors

Smoking behaviours were examined in seven studies [25–28, 32, 34, 47] (Supplementary Fig. S1H, available at *Rheumatology* online). Meta-analysis of four studies [27, 28, 34, 47] found strong evidence of a negative association between maternal prenatal smoking and JIA (pooled OR 0.70, 95% CI: 0.58, 0.84), with one study [27] also reporting a negative association between JIA and paternal prenatal smoking (adjusted OR 0.46, 95% CI: 0.21, 0.99). One study implied a dose-dependent relationship with higher levels of prenatal smoking decreasing the likelihood of JIA [47], this was unsupported by three further studies [25, 28, 32]. Meta-analysis of three studies [26–28] found little evidence of association between maternal smoking and JIA (pooled OR 0.75, 95% CI: 0.31, 1.81). Furthermore, one study [32] reported increased JIA risk associated with pre- and postnatal smoke exposure combined (adjusted OR 3.55, 95% CI: 1.38, 9.16). Two studies [27, 28] suggested that paternal (but not maternal or other relative) smoking inside the house is associated with decreased JIA risk (adjusted OR 0.26, 95% CI: 0.08, 0.81 and adjusted OR 0.64, 95% CI: 0.45, 0.92).

### Infection-related factors

Nineteen studies examined the association between JIA and infection-related factors [21–24, 26, 28, 42, 46, 48–58] (Supplementary Fig. S1I, available at *Rheumatology* online). Most data (including all pathogen-specific data) is from unadjusted analyses; only 8/19 studies reported any adjusted estimates [21–24, 26, 28, 56, 57]. There was little evidence that specific bacterial pathogens were associated with JIA. Parvovirus B19 was inconsistently associated with increased JIA risk [49–52]. There was little evidence of association between JIA and other viruses. One study [26] reported increased JIA risk associated with all cause infections (adjusted OR 1.92, 95% CI: 1.69, 2.18), which was supported by adjusted and unadjusted data from other studies [46, 49]. Similarly, antibiotic use was positively associated with JIA (unadjusted OR 1.53, 95% CI: 1.26, 1.85) [42]. However, unadjusted data suggests this is independent of age or frequency of exposure [58]. There was little evidence of association between JIA and mebendazole [56], palivizumab [57] or human papilloma virus vaccination [21–24].

### Environmental factors

Two studies [32, 59] examined factors related to the external environment (Supplementary Fig. S1J, available at *Rheumatology* online). Limited evidence suggests that higher maternal sunlight exposure during pregnancy is negatively associated with JIA [59]. Similarly, estimated childhood ultraviolet radiation exposure by quartile was also associated with decreased JIA risk in a dose-dependent manner (OR 0.19, 95% CI: 0.04, 0.85 for the highest quartile) [59]. With regard to pollutants, one study [32] reported that prenatal pollution exposure (adjusted OR 27.40, 95% CI: 6.85, 109.70) and exposure to second, but not third tertile, ozone in the first two years of life (adjusted OR 6.50, 95% CI: 2.15, 20.53 and adjusted OR 1.00, 95% CI: 0.54, 2.90, respectively) were risk factors for JIA.

## Discussion

### Summary of the evidence

The literature search strategy was designed to capture environmental factors in their broadest sense and identify all relevant studies regarding the association between these factors and JIA. A large number of articles were screened identifying 39 studies reporting 66 environmental factors for inclusion. Only eight environmental factors from ten studies were suitable for meta-analysis, highlighting the unmet need for replicated environmental research in JIA. Meta-analysis found strong evidence that CSD was associated with increased JIA risk whereas sibling status and maternal prenatal smoking appeared protective. There was limited evidence of increased JIA risk associated with post-term birth and SGA, and decreased JIA risk associated with maternal smoking, ≥3 siblings, birth weight (high or low) and preterm birth. No evidence of association between JIA and maternal age was found.

Of the 58 environmental factors not included in meta-analysis, some themes emerged. Consistent with sibling status, there was some evidence that increasing parity/number of prior births is protective of JIA whereas maternal fertility problems were associated with increased risk. However, data regarding number of siblings and birth order was conflicting. There was some evidence (largely from unadjusted data) of a positive association between JIA and both antibiotic exposure and early life infections but little evidence for specific pathogens. We found little evidence of association between JIA and maternal/child dietary factors, with the potential exception of fish consumption. Data regarding the association between JIA and breastfeeding status or duration of exclusive/total breastfeeding was highly conflicting. Low vitamin D levels have been causally implicated in multiple sclerosis risk [60]. However, only one study examined the association between vitamin D levels and JIA risk, finding no protective effect [29]. Other factors that influence vitamin D levels such as season of pregnancy/birth and maternal/offspring sunlight exposure showed conflicting findings.

### Interpretation of findings

JIA is considered a complex disease, influenced by genetic and environmental factors. The environmental factors identified in this review support several hypotheses explaining the pathological process leading to autoimmunity in JIA. The hygiene hypothesis proposed that the presence of siblings and subsequent early life exposure to infections was protective of atopy and autoimmunity due to effects on the immune repertoire [61, 62]. Accordingly, meta-analysis found decreased JIA risk in association with sibling status. In contrast, infectious agents have been proposed to contribute to autoimmunity through several mechanisms such as molecular mimicry, epitope spreading and bystander activation [63]. However, we found no consistent evidence of specific pathogen risk factors for JIA. More recently, there is a growing interest in the contribution of the microbiome to human health. An estimated 22–36% of inter-person microbiome variation is attributed to environmental influences [64], with early life environmental factors such as mode of delivery and breastfeeding influencing the microbiome later in life [65]. We identified CSD as a risk factor for JIA. CSD is postulated to affect the microbiome in multiple ways, including lack of exposure to vaginal flora, which begins at rupture of membranes (indeed, Kristensen and Henriksen reported increased JIA risk associated with elective but not acute CSD [36]), or routine administration of prophylactic intrapartum antibiotics which can pass to the foetus [66]. Changes in the microbiome may provide a unifying hypothesis to explain the association between CSD and JIA plus the weaker evidence of an association between antibiotic use and early life infections and JIA. However, CSD may be associated with JIA independent of any effects on the microbiome or may be mediating another exposure rather than itself being a primary risk factor for JIA. We were unable to determine a robust association between JIA and other perinatal/maternal factors known influence mode of delivery (e.g. pre-/post-term delivery, high/low birth weight, increasing maternal age and conception via assisted reproductive technology) in this review; however, we cannot exclude other indications for CSD as the primary exposure influencing JIA risk. Furthermore, dissecting direction of association from observational data is challenging and it remains possible that any association between JIA and infectious agents/the microbiome is in reverse; that JIA represents an end point of immune perturbation that also renders children more susceptible to infection and/or resultant exposure to antibiotics, or that active JIA disease/JIA treatments impact the microbiome.

Finally, environmental exposures have the potential to alter the epigenome and affect gene regulation [67]. Prenatal smoking has largely been associated with adverse offspring health and can alter immune responses [68], with altered DNA methylation a potential mediator [69]. Thus, our finding of a negative association with JIA was unanticipated. Explanations include specific effects of *in utero* smoke exposure on JIA risk or residual confounding, such as socioeconomic position or other measured/unmeasured confounders. However, the majority of studies of prenatal smoking did adjust for socioeconomic position. Furthermore, the effect of prenatal smoking on JIA risk is larger than for any other socioeconomic factor examined. Objective measures of prenatal smoking would be helpful to disentangle this association and limit potential reporting bias. Ultimately, the development of JIA is likely the result of complex and multifactorial immune interactions in genetically susceptible individuals, with many environmental factors making small but cumulatively important contributions to disease risk.

### Limitations of individual studies

The studies included in this review have some inherent limitations. Many studies had small sample size, thus imprecise findings cannot be assumed to represent no effect. Several studies examined multiple environmental factors and/or undertook subgroup analyses but did not account for findings potentially resulting from chance due to multiple testing. Fourteen studies diagnosed JIA based on International Classification of Diseases coding rather than physician-defined criteria, which may lead to misclassification bias. Confounder adjustment was variable, with 15/39 studies performing no adjustment. Although JIA can occur throughout childhood, only 1/11 cohort studies followed patients up to age 16 years. Substantial (>5%), or absent reporting of, loss to follow-up affected 8/11 cohort studies, potentially biasing estimates. With regard to case-control studies, 10/28 did not use community control subjects, limiting the generalizability of the results. Additionally, half of the case-control studies did not report the case and control response rate; therefore, we cannot exclude non-response bias from these studies.

### Strengths and limitations

Narrative reviews of environmental associations of JIA have recently been published [11, 12]. However, to our knowledge, this is the first study to systematically review environmental factors associated with JIA and attempt to quantify their effects. To its strength, this study conforms to PRISMA guidelines and was conducted in accordance with a pre-registered protocol [14]. We used a comprehensive and inclusive search strategy, composed with input from a literature searching specialist and implemented across multiple databases. To ensure the reliability of study selection, scoring and data extraction, all aspects involved a second, and where necessary a third, reviewer. The methodological quality of included studies was independently assessed using a validated scoring system. The included studies varied in quality; however, because JIA is rare we opted for inclusivity and no study was excluded based on quality assessment. Finally, to aid future research, in addition to the data reported in the main text, we have made our full dataset available.

The main limitation of this review is bias due to heterogeneity, which has arisen for several reasons. Firstly, the diagnostic criteria for JIA have evolved over the review period [3]. Secondly, individual studies varied in their inclusion/exclusion of specific JIA subtypes. Thirdly, because the review period spans 45 years, it is likely that social practices, and thus environment influences, have changed. Collectively, historic JIA cohorts may differ from those diagnosed using more recent criteria. However, two-thirds of studies were published in the past decade, and half within the past five years. Despite searching multiple databases and hand-searching bibliographies of excluded review articles, the breadth of this review means we may not have captured all relevant studies. Additionally, we excluded studies where we could not extract JIA-specific data from composite outcomes; such data may be informative. Due to resource constraints, only studies in English were included; however, we felt it unlikely that this led to omission of major relevant studies. We were unable to formally assess potential publication bias using funnel plots due to the small numbers of studies investigating each environmental factor. Bias may also result from studies rarely adjusting for the same factors (if at all) and the different lengths of follow-up between studies. Environmental factors across studies were seldom identified, defined and/or measured in the same way, or could be transformed into comparable measures. This resulted in sufficient data to assess only eight factors using meta-analysis. Of these, three included only two studies. Accordingly, for most meta-analyses the pooled estimates were imprecise (encompassing weak evidence of both positive or negative effects) and should be interpreted with caution rather than taken as evidence of no effect. Several studies were excluded where the outcome was JIA course/severity rather than incidence. Whether environmental factors for disease course/severity overlap with those for incidence is an important research question but is outside the scope of this review. Finally, because this review includes only observational studies, the inevitable question remains as to whether our findings represent correlation or causation.

### Implications of findings

Identifying robust risk or protective factors associated with JIA has enormous capability to alter patient counselling, aid risk stratification and inform future (causal) research. Furthermore, environmental factors may be modifiable with implications for population JIA risk reduction. The identification of risk (CSD) and protective (sibling status and prenatal smoking) factors associated with JIA necessitates further study into causality and mechanism, and the identification of other putative risk factors (e.g. antibiotics) highlights areas for research priority. The inability to pool the majority of environmental data underscores the need for research reproducibility and standardization of study design. Validating the findings of this review and identifying novel risk/protective factors will require further studies in large populations and likely require international collaboration and co-operation to align this work. Close consideration should be given to the measurement of and adjustment for confounders. Observational studies need to be integrated with mechanistic studies and other data modalities (such as genomic or microbiomic data) to attempt to delineate correlation from causation and improve our understanding of JIA aetiopathogenesis.

## Conclusions

This review highlights the plethora of environmental research undertaken in JIA over the last five decades and the challenges posed by using data from historical cohorts. The environmental factors identified here will assist in planning future studies to probe the extent of these associations and understand JIA aetiopathogenesis more broadly, which will ultimately translate into patient benefit.

## Supplementary Material

keab627_supplementary_data
