# Supplementary material for: Moving from nature to nurture: a systematic review and meta-analysis of environmental factors associated with juvenile idiopathic arthritis
Source: Rheumatology (Oxford). 2021 Aug 11;61(2):514–30. doi: 10.1093/rheumatology/keab627 (PMC8824412; doi:10.1093/rheumatology/keab627)
Supplement: keab627_supplementary_data [file keab627_supplementary_data.zip › rhe-21-1326-File004.docx]

**Supplementary Data S1. Supplementary methods**

Most of the studies examining the association between JIA and maternal age reported the OR of JIA according to different maternal age brackets with reference to a baseline, rather than a continuous measure. In order to include these studies in a quantitative analysis, ordinal categorical data was combined into a single linear effect estimate using generalised least squares for trend estimation^1^ (GLST) implemented using the *glst* module in Stata^2^. GLST produces a ratio effect measure (in this case odds ratio) for a unit change in the exposure variable on the continuous scale by estimating the trend across the odds ratios for the exposure categories.

The information required by GLST is a) the number of exposed and unexposed participants in each age category, b) the mean age in each category and c) the ORs (expressed as beta-coefficients) and standard errors for each non-reference maternal age category. For Thorsen *et al*, the number of exposed and unexposed events in each category was not recorded, therefore we considered maternal age to be normally distributed and derived the counts as follows. The standard deviation was derived for each group of participants (oligoarticular JIA, polyarticular and healthy controls) from the reported interquartile range (IQR), where IQR is approximately 1.35 standard deviations. Z scores were approximated by determining the number of standard deviations between the median age and the upper categorical bounds (25 years and 35 years). Using a cumulative density function, we approximated the proportion of the total number participants falling within each category. The number of oligoarticular JIA and polyarticular JIA participants in a given category were summed to provide the total number of JIA cases within each category. The mean for each category (bounded and unbounded) was estimated using the method proposed by Chene and Thompson^3^. For each category, the change in the exposure from that of the reference category is calculated as difference from zero.

Following data preparation the GLST estimation method was then applied using the *glst* command in STATA. In brief, the expected number of exposed and unexposed participants in each category is estimated from the adjusted OR and number of participants in each category using a fitting algorithm. A variance-covariance matrix is then estimated from the fitted values. These estimates are then used to approximate the odds ratio per unit change in maternal age. Since the resulting estimates are beta-coefficients, these are back-transformed (exponentiated) to represent the OR on the continuous scale.

References

1. S Greenland and MP Longnecker. Methods for trend estimation from summarized dose-response data, with applications to meta-analysis. Am J Epidemiol. 1992;135(11):1301-9. doi: 10.1093/oxfordjournals.aje.a116237

2. N Orsini, R Bellocco and S Greenland. Generalized least squares for trend estimation of summarized dose-response data. Stata Journal. 2006;6(1):40-57. doi:

3. G Chene and SG Thompson. Methods for summarizing the risk associations of quantitative variables in epidemiologic studies in a consistent form. Am J Epidemiol. 1996;144(6):610-21. doi: 10.1093/oxfordjournals.aje.a008971


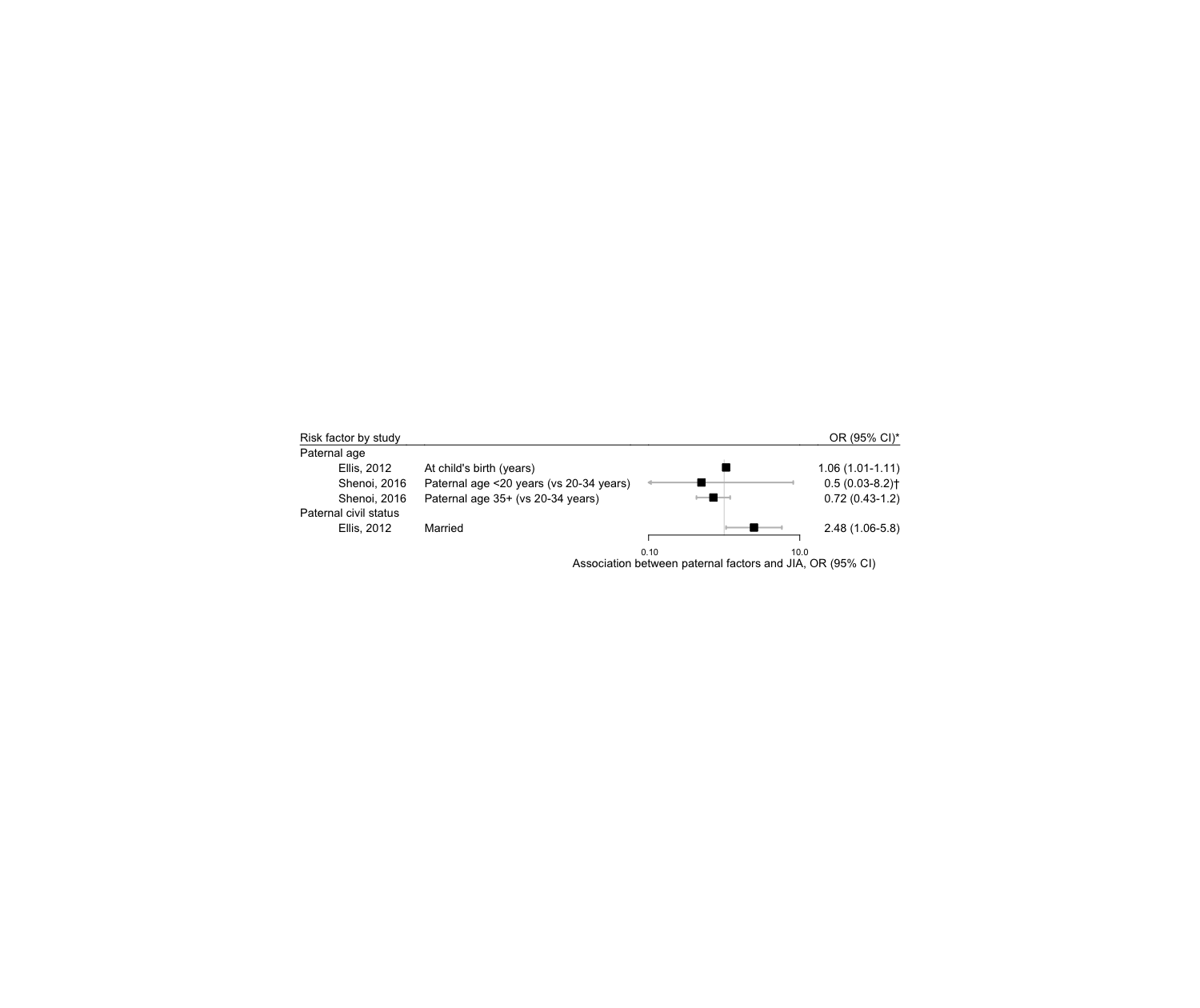


B


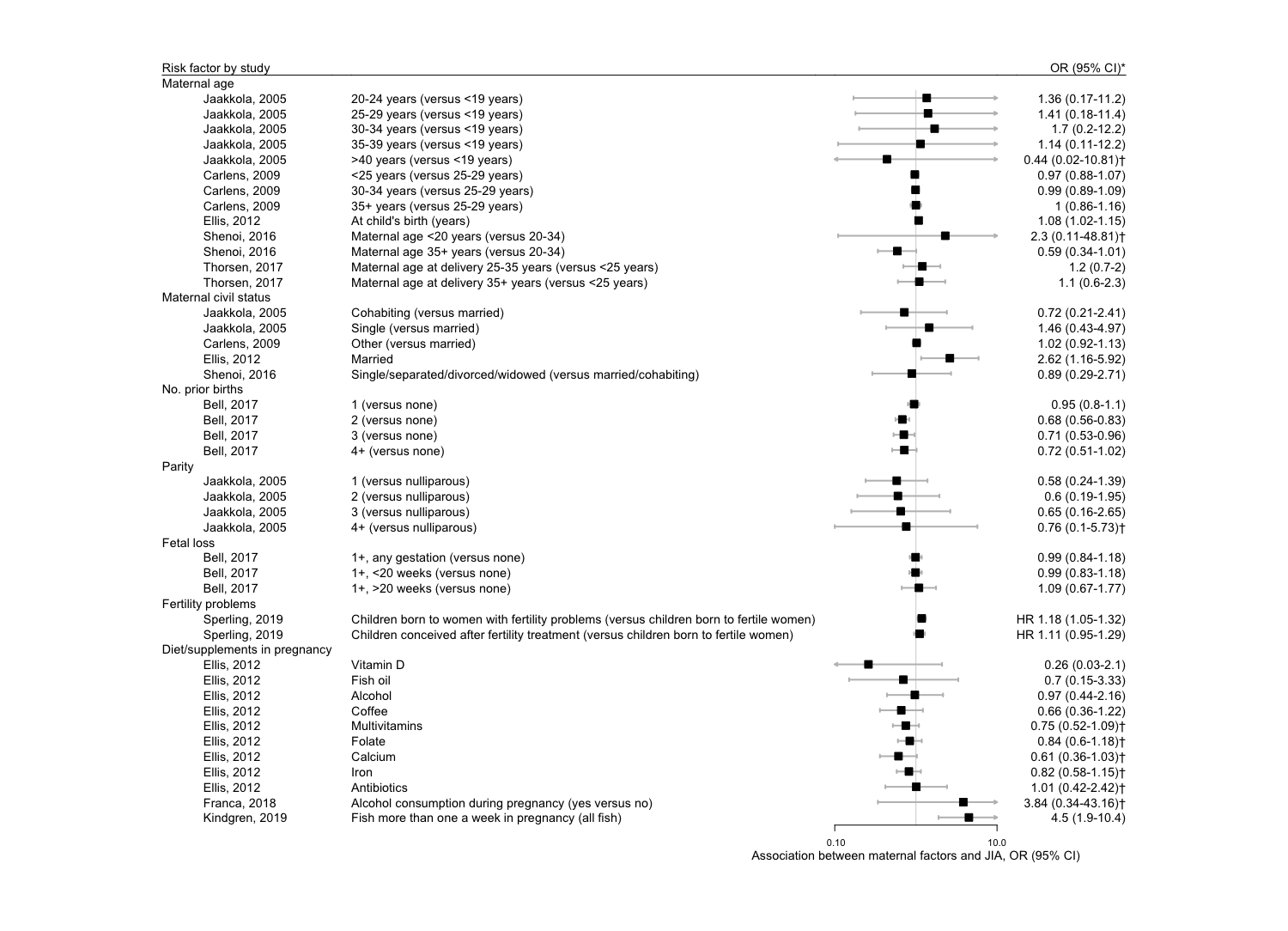


A

**Supplementary Figure S1: Association between JIA and maternal (A), paternal (B), perinatal (C), early life (D), diet (E), living environment (F), SES (G), smoking (H), infection-related (I) and environmental (J) factors**. A single point estimate with 95% CI has been collated for each variable (see Methods).*all reported estimates are adjusted OR (95% CI) unless otherwise stated. †denotes derived univariate OR (95% CI), ‡denotes study reported estimate (95% CI). AGA, average for gestational age; CI, confidence interval; CMV, cytomegalovirus; EBV, Epstein-Barr virus; HR, hazard ratio; Ig, immunoglobulin; IRR incidence rate ratio; LGA, large for gestational age; O3, ozone; OR, odds ratio; SGA, small for gestational age; UVR, ultraviolet radiation.


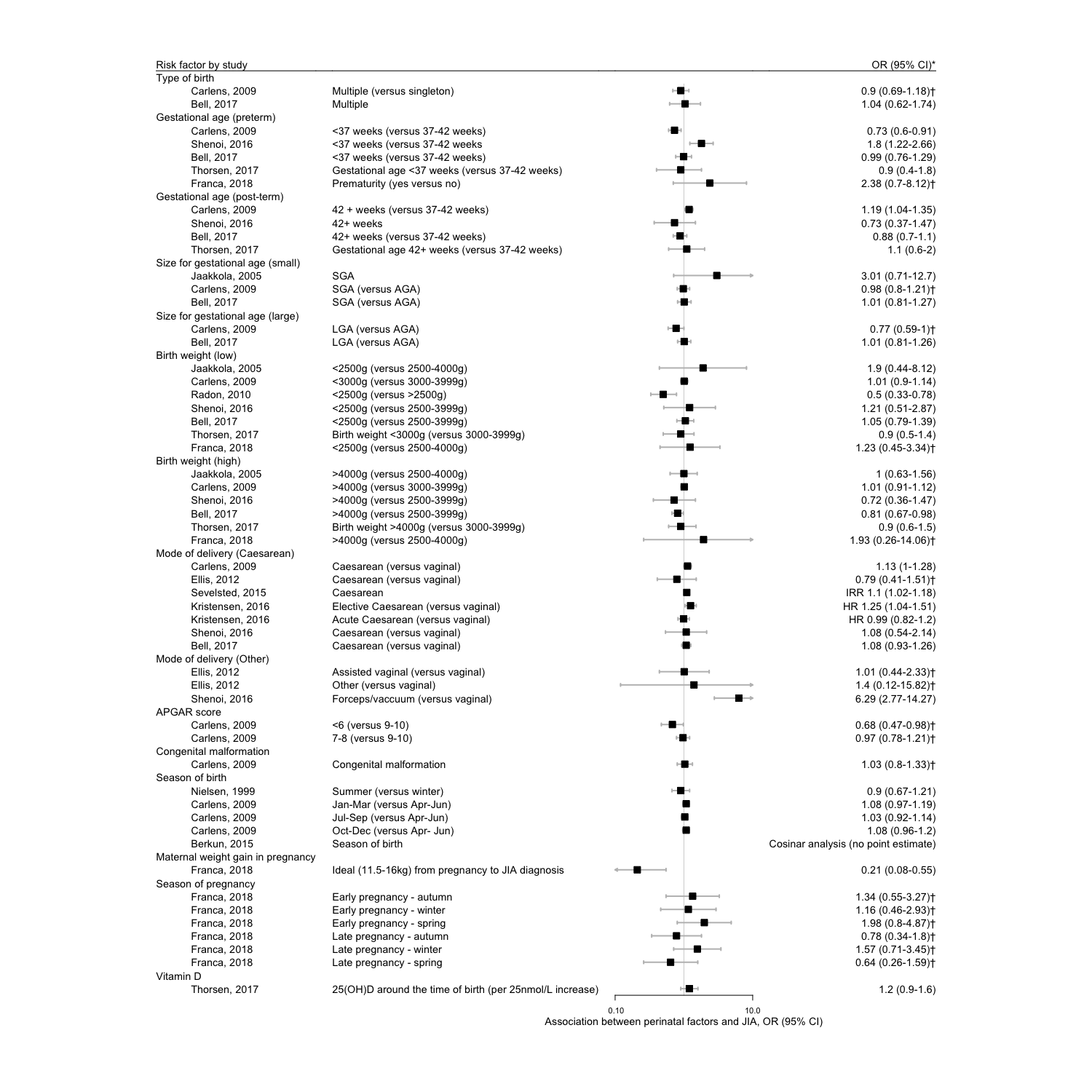


C


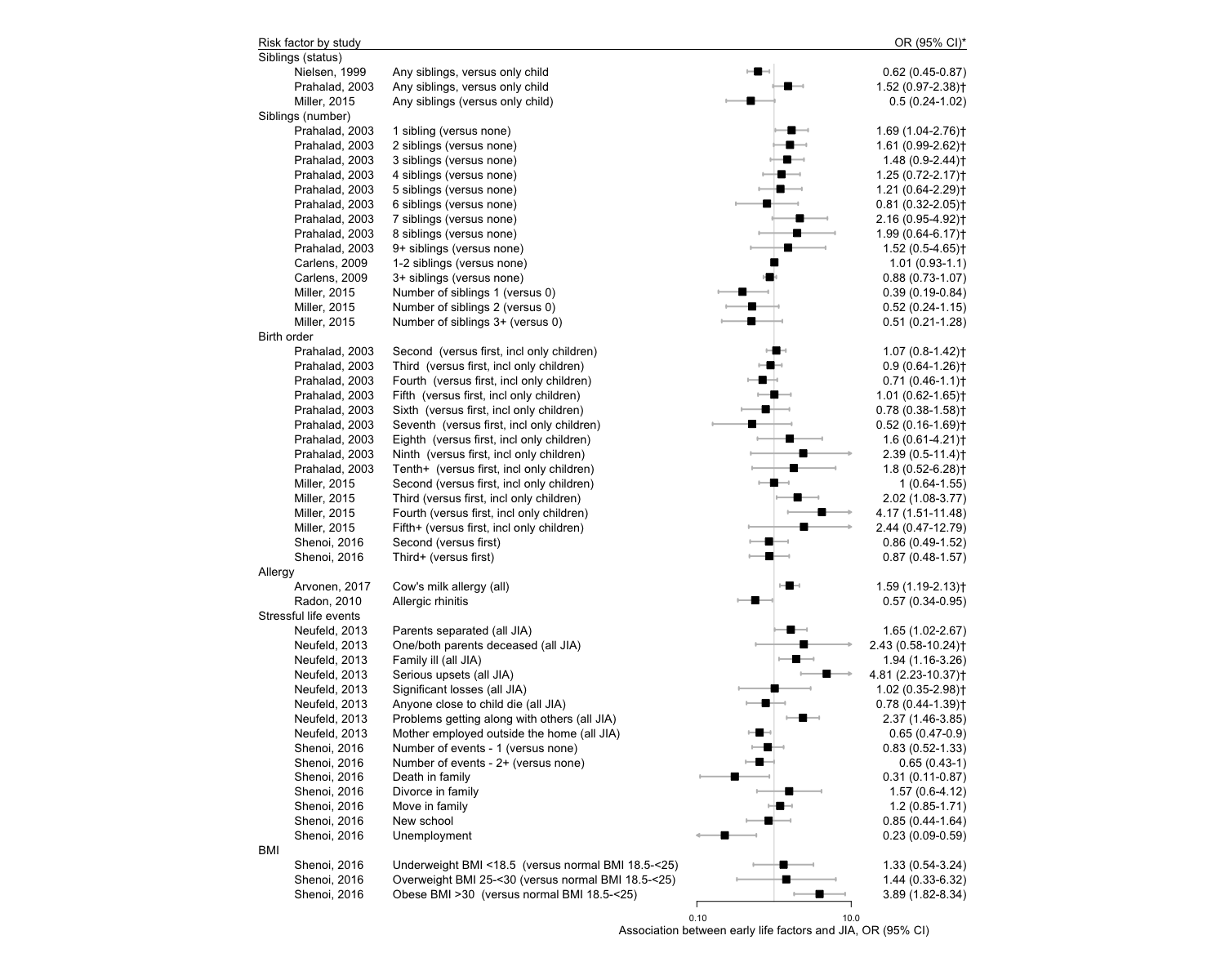


D


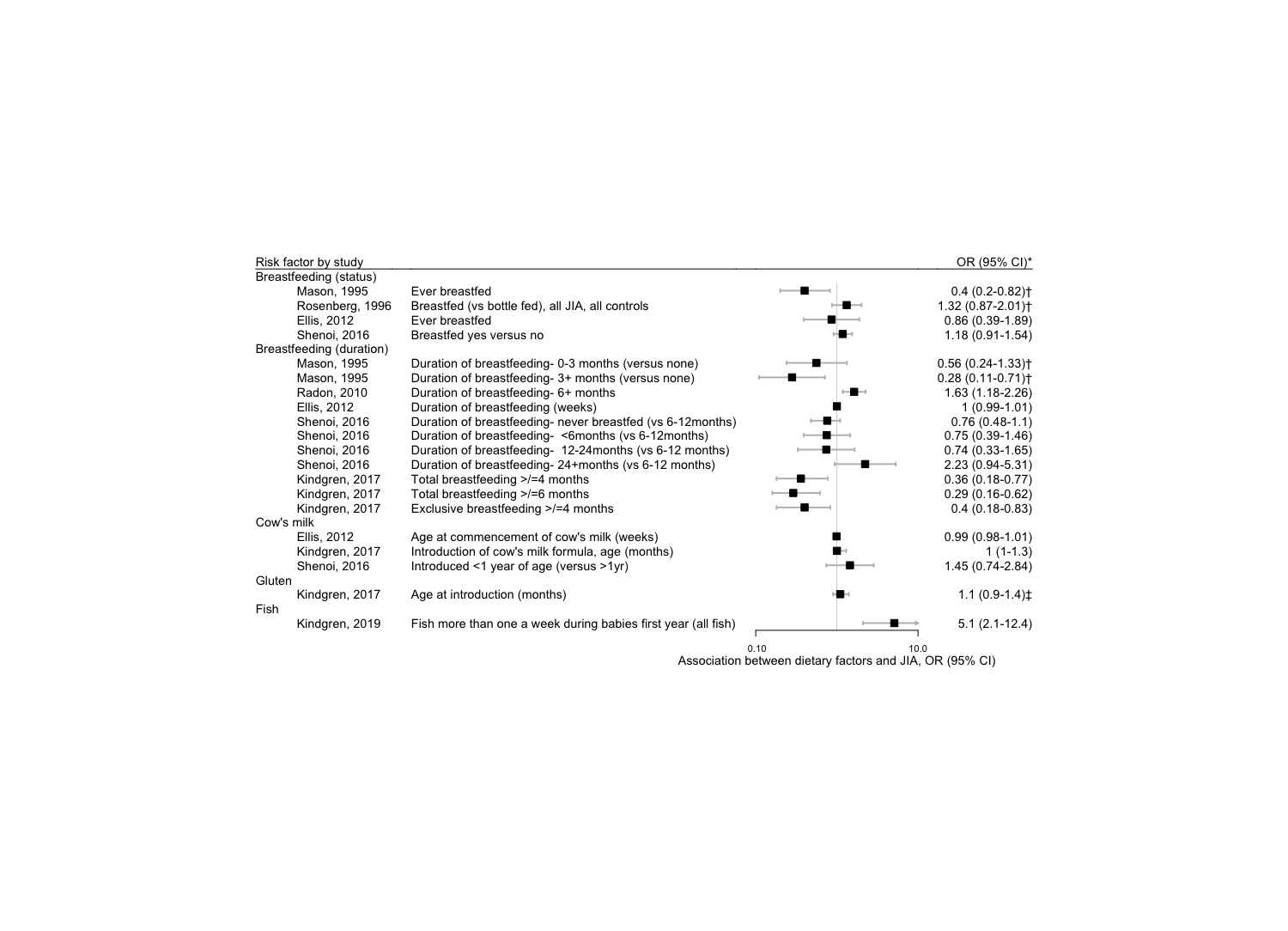


E


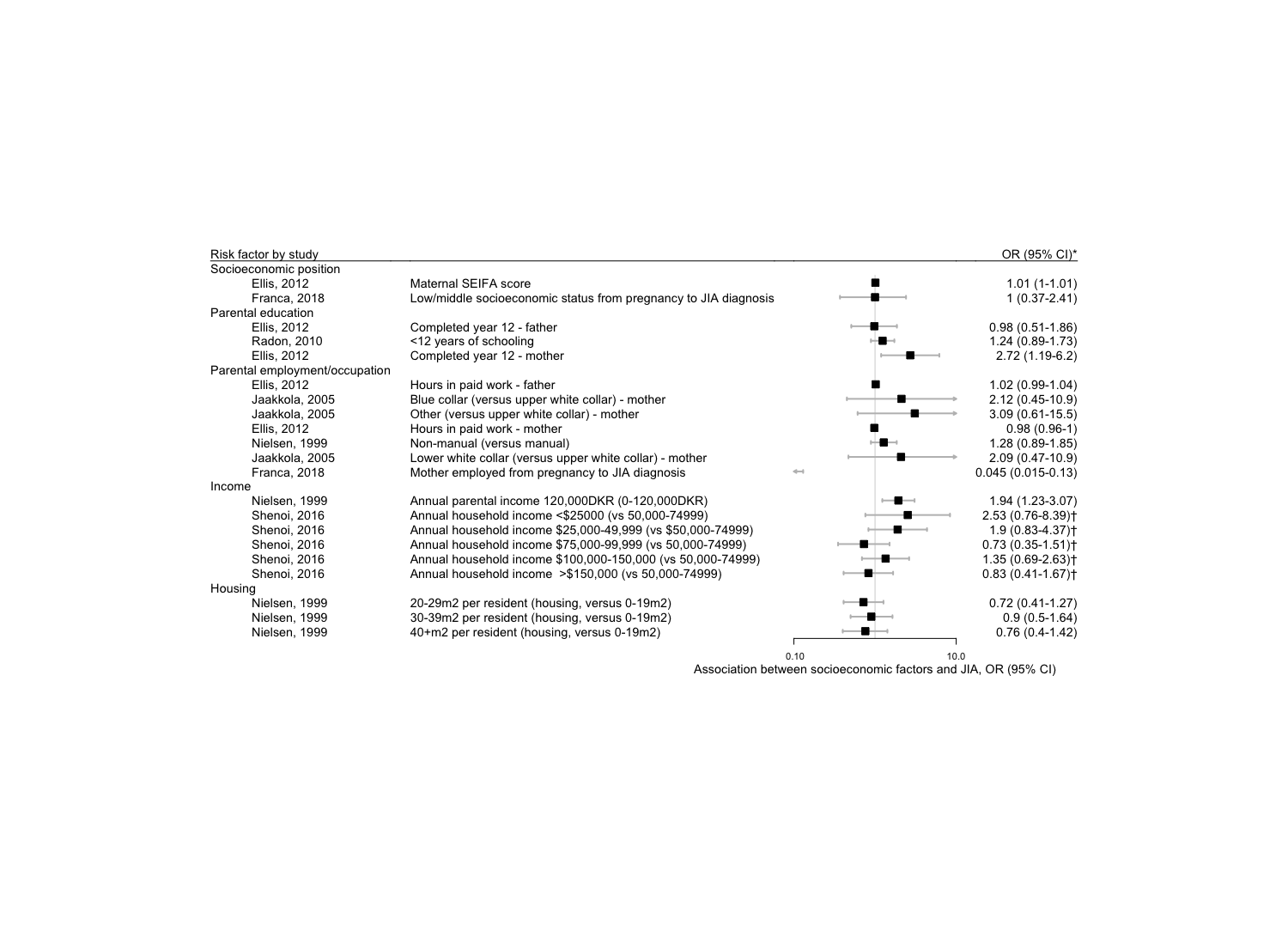


G


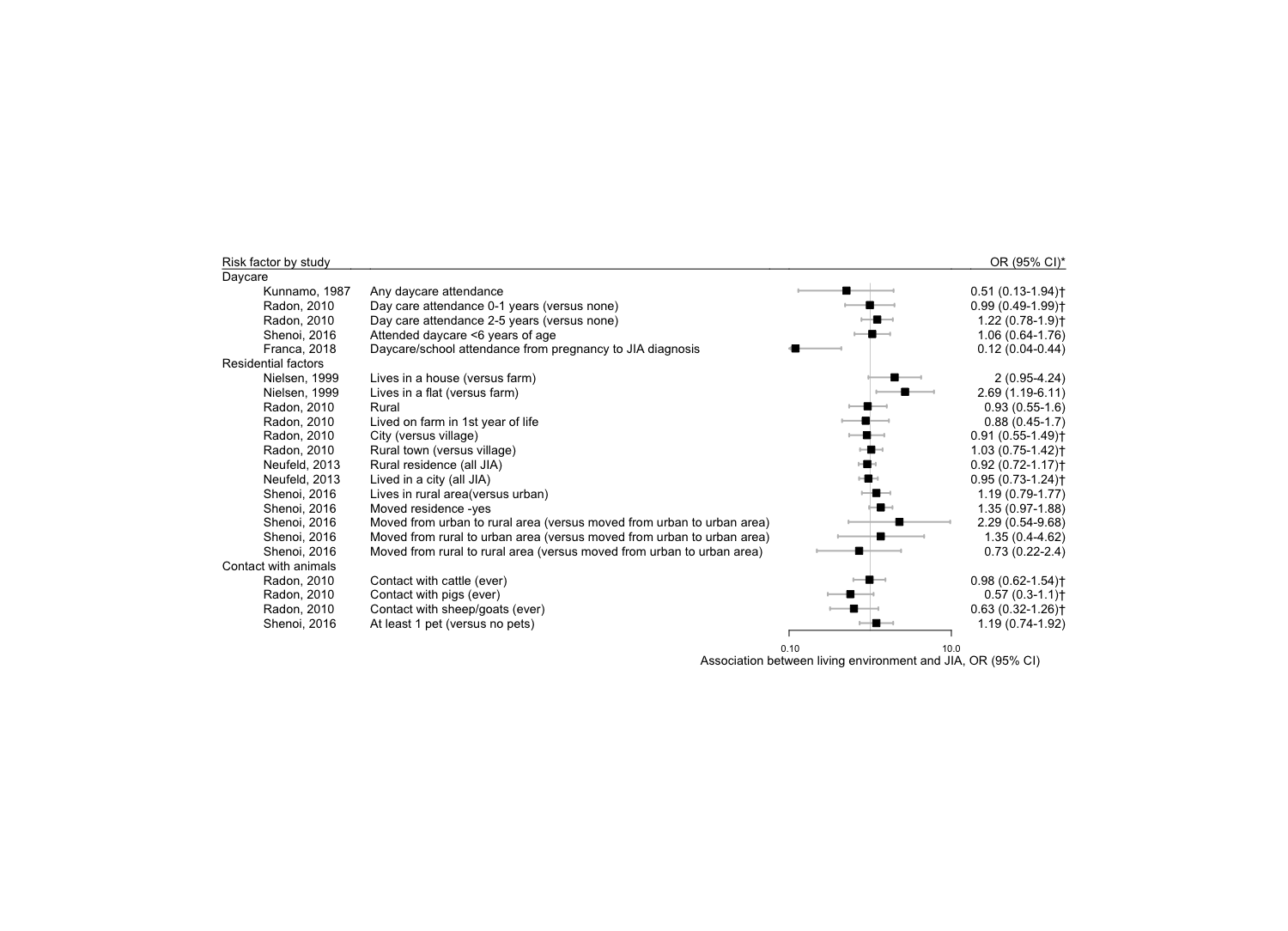


F


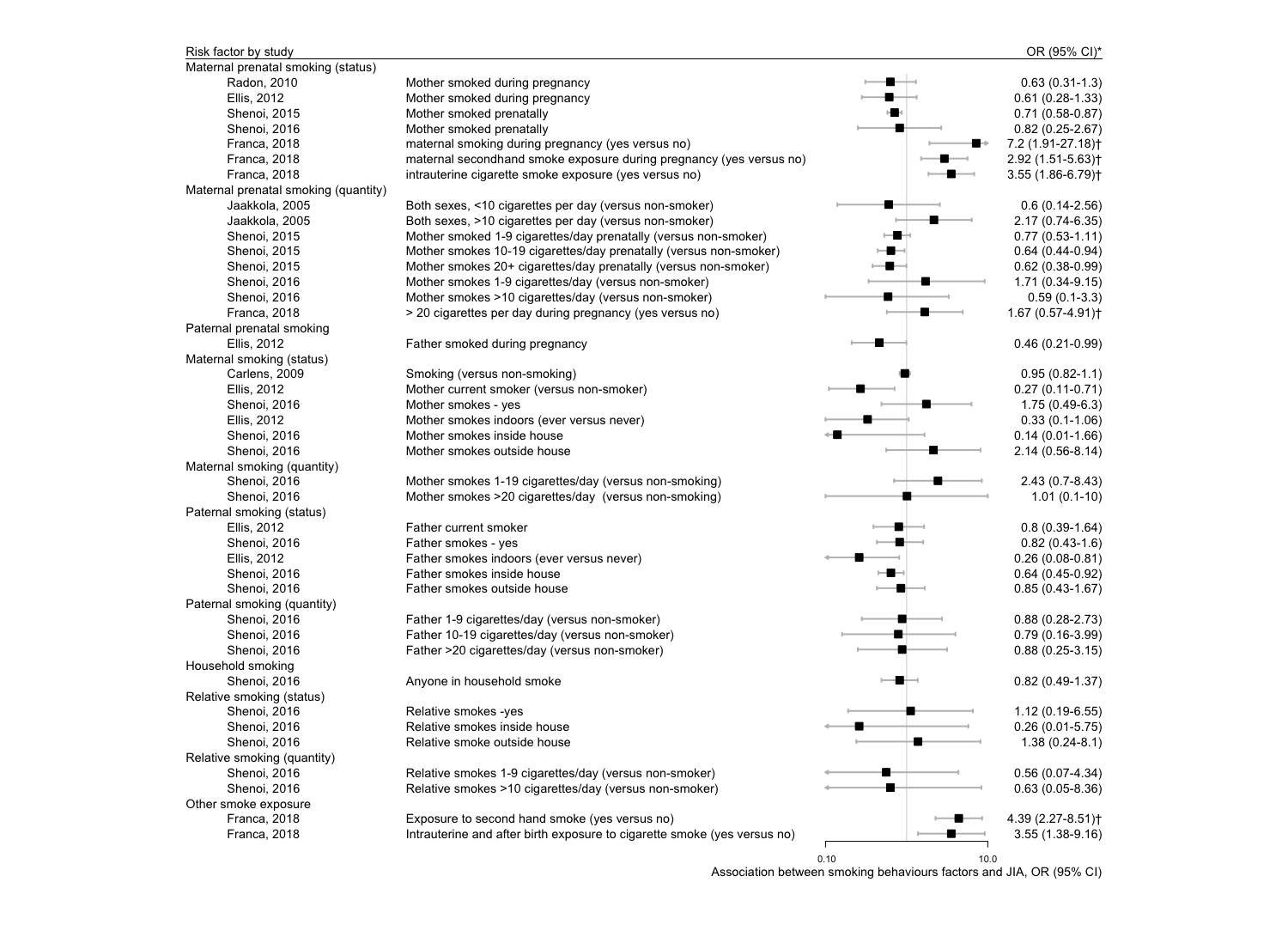


H

I


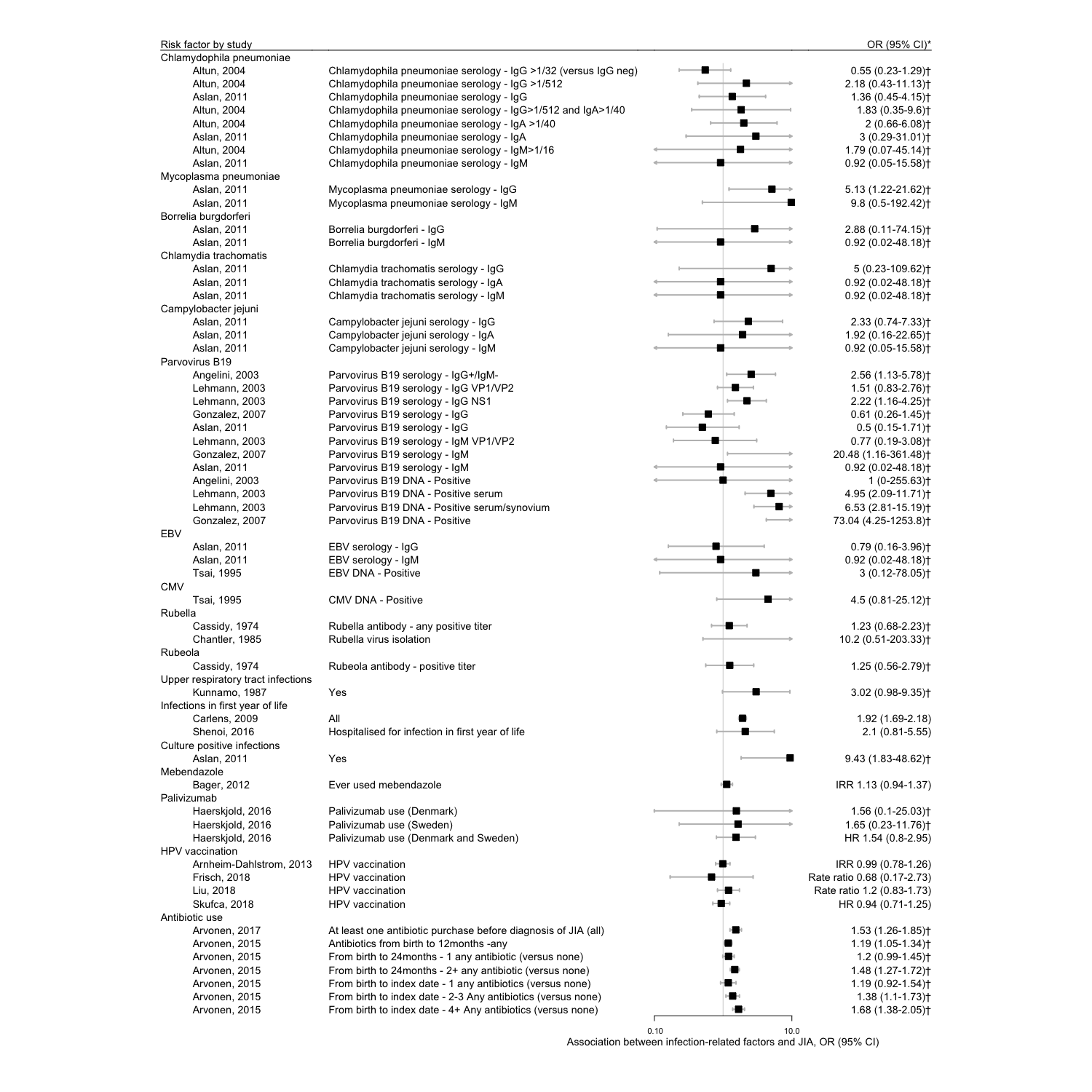


.


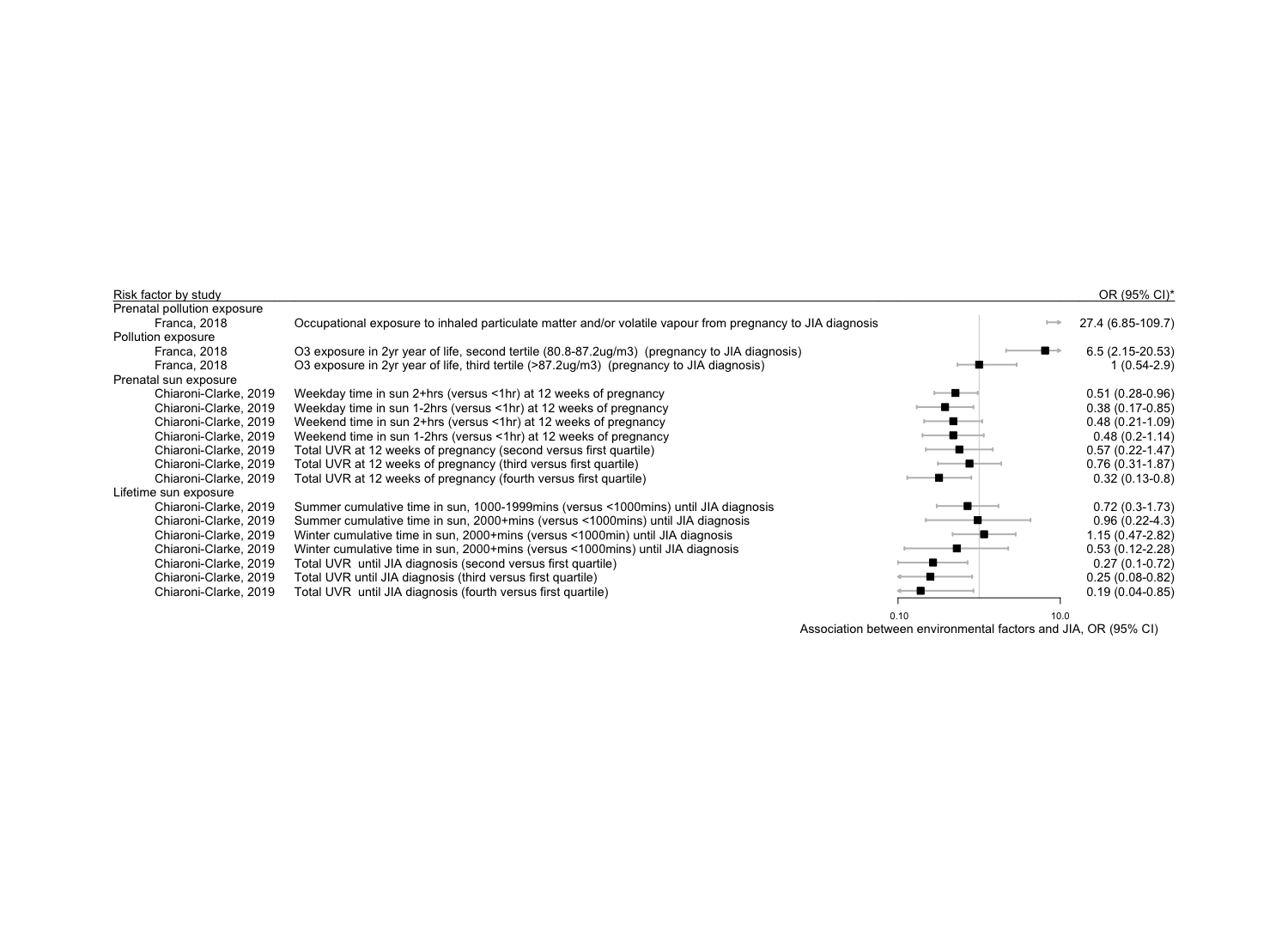


J
